# Pulsed Field Ablation for Atrial Fibrillation: Contemporary Clinical Evidence and Real-World Experience in Redo Ablation

**DOI:** 10.3390/jcm15041647

**Published:** 2026-02-22

**Authors:** Ioanna Koniari, Eleni Artopoulou, Scott Gall, Gavin S. Chu, Rafail Koros, Maria Bozika, Kassiani-Maria Nastouli, Georgios Leventopoulos, Shajil Chalil, Aruna Arujuna

**Affiliations:** 1 Department of Cardiology, University Hospital of Patras, 26504 Patras, Greece; 2 Liverpool Centre for Cardiovascular Science, Liverpool L14 3PE, UK; 3 KreiskrankenhausCalw, AkademischesLehrkrankenhaus Universität Tübingen, 72074 Tübingen, Germany; 4 Lancashire Cardiac Centre, Blackpool Teaching Hospitals NHS FT, Blackpool FY3 8NR, UK

**Keywords:** pulsed field ablation, atrial fibrillation, pulmonary vein isolation, catheter ablation, electroporation

## Abstract

Atrial fibrillation (AF) is the most common prevalent sustained arrhythmia and is associated with stroke, heart failure, and impaired health-related quality of life. Due to the complexity of the initiation and the persistence of AF, the pulmonary vein isolation (PVI) using thermal or laser energy is the most commonly applied ablation strategy. However, these thermal ablation modalities have several limitations, including a substantial risk of AF recurrence and collateral damage to tissues adjacent to the heart. Pulsed field ablation (PFA) is a novel non-thermal ablation technique in which high-voltage electric fields deliver short pulses, selectively affecting cardiomyocyte cell membranes. PFA has the potential to create myocardial lesions with minimal harm to non-cardiac tissues. Clinical studies have evaluated the safety and efficacy of PFA, examining its ability to prevent AF recurrence and its impact on surrounding structures, often in comparison with conventional PVI approaches. In this review, PFA clinical studies are discussed as well as our experience with the PFA use for redo atrial fibrillation ablation cases.

## 1. Introduction

Atrial fibrillation (AF) is the most common sustained cardiac arrhythmia affecting 1–2% of the general population. The onset of AF is associated with an increased risk of stroke and the development of heart failure (HF), as well as impaired functional capacity and quality of life [1]. According to the European Society of Cardiology guidelines, catheter ablation of AF is recommended for symptomatic patients who are refractory to optimal medical therapy or have concomitant tachycardia-induced cardiomyopathy or heart failure (HF) [2,3]. Due to the complexity of AF pathogenesis, pulmonary vein (PV) ectopic foci are believed to play a key role in initiating and sustaining AF. Consequently, pulmonary vein isolation (PVI) using radiofrequency (RF), thermal, or laser energy constitutes the cornerstone of AF ablation therapy [1]. However, current ablation techniques have several limitations, whereas PV reconnection occurs in 20% of patients post initial PVI procedure [4]. Additionally, thermal ablation technologies can cause significant collateral injury to structures adjacent to the heart, particularly the esophagus or phrenic nerve such as atrio-esophageal fistula or phrenic nerve paralysis [2,5].

**Figure 1:**
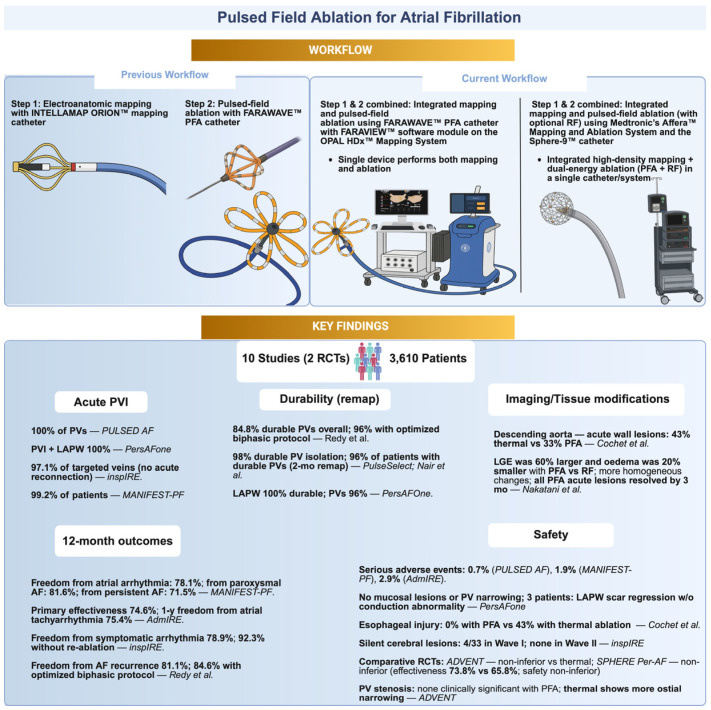
Technological workflow evolution and key clinical outcomes of PFA for atrial fibrillation. The upper panel illustrates the transition from a sequential workflow, electroanatomic mapping followed by pulsed field ablation using separate catheters, to integrated systems enabling combined high-density mapping and ablation with a single catheter. The lower panel summarizes key findings from major clinical studies, including acute pulmonary vein isolation rates, lesion durability on remapping, imaging-based tissue effects, 12-month arrhythmia outcomes, and safety endpoints. Figure created in BioRender. Bozika, M. (2025) https://BioRender.com/6xb5zp7 (accessed on 2 December 2025).

One of the most recent advances in the catheter ablation area is pulsed field ablation (PFA), a non-thermal ablation modality that uses high-voltage electric pulses delivered between electrodes to create pores in cardiomyocyte cell membranes in a highly selective, tissue-specific manner. This targeted electroporation leads to irreversible myocardial cell death while minimizing damage to non-cardiac tissues including the esophagus, nerves and vessels [6]. Importantly, PFA does not disrupt the extracellular matrix, thereby preserving tissue planes and reducing risks such as atrio-esophageal fistula associated with RF ablation. PFA procedures are usually performed under general anesthesia with intubation, or under deep sedation, to minimize patient discomfort and muscle contractions, leading to improved procedural tolerance and efficacy [1]. Using a non-thermal mechanism of cellular injury is expected to improve the safety and efficacy of cardiac ablation. However, it remains uncertain whether PFA can achieve long-term freedom from AF recurrence (Figure 1).

## 2. Clinical Studies of PFA

Astudy by Reddy et al. [7] used data pooled from threetrials (IMPULSE [A Safety and Feasibility Study of the IOWA Approach Endocardial Ablation System to Treat Atrial Fibrillation], PEFCAT [A Safety and Feasibility Study of the FARAPULSE Endocardial Ablation System to Treat Paroxysmal Atrial Fibrillation], and PEFCAT II [Expanded Safety and Feasibility Study of the FARAPULSE Endocardial Multi Ablation System to Treat Paroxysmal Atrial Fibrillation]) to evaluate the 1-year clinical outcomes of PFA, including early and late onset safety endpoints, durability and freedom of cardiac arrhythmias. In total the trials included 121 patients where a 12-F over-the-wire PFA catheter was used in order to achieve PVI [7,8]. In the IMPULSE trial, 40 patients underwent PFA with monophasic waveforms between 900 and 1000 V under general anesthesia. In the PEFCAT and PEFCAT II trials biphasic waveforms between 1800 and2000 V were used under sedation, which was well tolerated. No reconnection was observed after a 20 min waiting period or adenosine testing. After 75–90 days from the procedure, 110 patients underwent remapping of the left atrium where a multielectrode catheter was used to evaluate possible reconnection of pulmonary veins.The follow-up was 1 year by clinical visit, trans-telephonic monitor, and 24 h Holter to evaluate the recurrence of atrial fibrillation. Despite the occurrence of two cases of cardiac tamponade, the rate of primary safety events was low (2.5%), whereas no evidence of esophageal injury, phrenic nerve lesion, or brain lesions after ablation were observed. In the following three months, left atrium remapping demonstrated durable PVI in 84.8% of PVs. This percentage included 45.2% of PVI in 11 patients treated with monophasic energy, 83.6% in patients treated with biphasic energy and 96% in patients treated with biphasic energy with optimized biphasic protocol. Across 12 months of rhythm surveillance (clinical review, transtelephonic monitoring, and Holter recordings), Kaplan–Meier freedom from recurrent AF was 81.1% ± 3.8%, rising to 84.6% ± 6% among patients treated with an optimized biphasic protocol [1,7,8].

The pivotal PULSED AF study (Pulsed Field Ablation to Irreversibly Electroporate Tissue and Treat AF) was a prospective, global, multicenter, nonrandomized, paired single-arm study evaluating a bipolar, biphasic PFA system for PVI in symptomatic, antiarrhythmic drug-refractory paroxysmal (n = 150) and persistent (n = 150) AF [9]. Follow-up included weekly transtelephonic transmissions, scheduled ECGs at 3, 6, and 12 months, and 24 h Holter monitoring at 6 and 12 months, with the first 3 months treated as a blanking period [9]. Effectiveness was assessed by the absence of acute procedural failure and subsequent arrhythmia recurrence or escalation of antiarrhythmic therapy, while safety focused on serious device- or procedure-related adverse events. Acute PVI was achieved in 100% of PVs. Major complications were uncommon (estimated 0.7%), and no clinically evident phrenic nerve injury, esophageal injury, or pulmonary vein injury was reported. The observed effectiveness was broadly comparable to contemporary outcomes with thermal ablation approaches [10,11], and quality-of-life measures improved significantly from baseline [12,13,14].

In a mechanistic imaging study, Cochet et al. compared extra-atrial tissue effects after PVI performed with PFA versus thermal energy in 41 patients with paroxysmal AF [15]. Cardiac magnetic resonance imaging was obtained before ablation, within 3 h after the procedure, and again at 3 months. Among patients treated with radiofrequency or cryoballoon ablation, acute esophageal lesions were identified in 43%, whereas no esophageal injury was seen after PFA; phrenic nerve injury was not documented in either group. Acute changes in the descending aorta were observed after both modalities (43% thermal vs. 33% PFA), with resolution on follow-up imaging. In a subsequent analysis, Nakatani et al. described more homogeneous atrial tissue changes after PFA, with larger acute late-gadolinium enhancement volumes but less edema compared with radiofrequency, and fewer persistent lesions at 3 months—consistent with limited chronic fibrotic remodeling [16].

PersAFOne (PulsedFields for Persistent Atrial Fibrillation) evaluated biphasic, bipolar PFA delivered via a multispline catheter for combined PVI and left atrial posterior wall (LAPW) ablation, guided by intracardiac echocardiography; a focal PFA catheter was used for cavotricuspid isthmus ablation, without additional esophageal protection [17]. Invasive reassessment within 2–3 months was used to test lesion durability. In the initial cohort (n = 25), acute PVI and LAPW isolation were achieved in all patients, and cavotricuspid isthmus block was obtained in all 13 patients requiring this lesion set. Post-procedure endoscopy and computed tomography showed no mucosal injury and no pulmonary vein narrowing. Remapping demonstrated durable isolation in 96% of pulmonary veins (82/85) and 100% of posterior wall targets (21/21), with localized posterior wall scar regression in three patients without reconduction

The inspIRE study (Study for Treatment of Paroxysmal Atrial Fibrillation) assessed a fully integrated biphasic PFA platform incorporating a variable-loop circular catheter and a 3D mapping system in drug-refractory paroxysmal AF [18]. Among 226 treated participants (Wave I: 40; Wave II: 186), the primary safety outcome was early major adverse events, while effectiveness incorporated confirmed PVI and freedom from documented atrial arrhythmia at 12 months. In Wave I, no esophageal thermal lesions or pulmonary vein stenosis were observed, although silent cerebral lesions were detected on MRI in 4 of 33 scanned participants. No primary adverse events were reported in Wave II. Acute PV isolation without reconnection was achieved in 97.1% of targeted veins, and 12-month freedom from symptomatic atrial arrhythmia and repeat ablation was 78.9% and 92.3%, respectively. MANIFEST-PF (Multi-National Survey on the Methods, Efficacy, and Safety on the Post-Approval Clinical Use of Pulsed Field Ablation) was a retrospective, multinational registry reporting post-approval PFA practice across 1568 patients with paroxysmal or persistent AF [19].PVI was achieved in 99.2% of cases. Over a median follow-up of 367 (289–421) days, the Kaplan–Meier estimate for freedom from atrial arrhythmia at 1 year was 78.1% (95% CI, 76.0–80.0%), with higher effectiveness in paroxysmal than persistent AF (81.6% vs. 71.5%; *p*= 0.001). Acute major adverse events occurred in 1.9% of patients, with additional latent events captured in the composite safety outcome.

The ADVENT was a multicenter, randomized, single-blind trial comparing PFA with conventional thermal ablation (radiofrequency or cryoballoon) in drug-refractory paroxysmal AF [20]. A total of 607 participants were treated with PFA (n = 305) or thermal ablation (n = 302). Post-procedure management included guideline-directed anticoagulation and class I/III antiarrhythmic therapy (amiodarone excluded). Patients were followed for 12 months using 72 h Holter monitoring and transtelephonic ECG transmissions; CT or MRI was used to evaluate pulmonary vein anatomy, and selected patients underwent brain MRI. PFA met noninferiority criteria versus thermal ablation for the composite efficacy endpoint and for the composite safety endpoint over the first year. Clinically significant pulmonary vein stenosis was not observed in either arm; however, imaging-defined ostial narrowing occurred more frequently after thermal ablation, particularly radiofrequency.

AdmIRE (Assessment of Safety and Effectiveness in Treatment Management of Atrial Fibrillation With the Biosense-Webster Irreversible Electroporation Ablation System), is a multicenter, single-arm pivotal study evaluating a variable-loop circular PFA catheter coupled to a multichannel generator and a mapping system that provides real-time feedback on catheter–tissue proximity [12,21]. The trial enrolled 277 adults (18–75 years) with symptomatic, drug-refractory paroxysmal AF undergoing first-time PVI, with uninterrupted anticoagulation required pre-procedure and continued for at least 2 months afterward [21]. The primary safety endpoint captured serious device- or procedure-related complications including tamponade/perforation, thromboembolism, atrio-esophageal fistula (through 90 days), and pulmonary vein stenosis during follow-up. Effectiveness included acute entrance block in all PVs and 12-month freedom from documented atrial tachyarrhythmias without repeat ablation [21]. The primary adverse event rate was 2.9% (8 of 272), most commonly pericardial tamponade, and the primary effectiveness endpoint was 74.6%; 1-year freedom from atrial tachyarrhythmia was 75.4%. Procedure and fluoroscopy times were short, and quality-of-life improved as early as 3 months post-ablation [21].

SPHERE Per- was a randomized, single-blind, noninferiority trial in persistent AF comparing an investigational large-tip catheter capable of both pulsed-field and radiofrequency energy delivery with a conventional radiofrequency ablation system (n = 420) [22]. The primary effectiveness endpoint incorporated acute procedural success and absence of arrhythmia recurrence, cardioversion, drug initiation/escalation, or repeat ablation beyond a 3-month blanking period; the primary safety endpoint was freedom from serious procedure- or device-related adverse events. Noninferiority was achieved for effectiveness (73.8% vs. 65.8%; *p*< 0.0001 for noninferiority) and for safety, with few major complications in either arm [22].

Additional early experience with a circular PFA catheter (PulseSelect) was presented by Nair et al. at the Asia Pacific Heart Rhythm Society annual meeting (28 September 2024) [23]. In 25 patients with paroxysmal or persistent AF, PVI was performed with intracardiac echocardiography and 3D mapping guidance without fluoroscopy. Invasive assessment immediately after ablation and again at 2 months suggested high lesion durability, with 98% of PVs remaining isolated and 96% of patients maintaining complete PVI. No procedure-related adverse events were reported over a mean follow-up of 74 ± 14 days [23]. All the aforementioned studies and respective endpoints and outcomes are summarized in Table 1.

## 3. Safety and Efficacy of PFA for Redo AF Ablation Procedures: Case Series of Lancashire Cardiac Centre and University Hospital of Patras

Before the introduction of PFA technology, recurrence of atrial fibrillation after the index procedure with thermal energy ablation either with one shot technique (cryoballoon or laser ) or classic point–point RF application was performed with the guidance of available mapping systems (Ensite, CARTO, Rhythmia/Opal HDx) to recognizeelectrical conduction into pulmonary veins and further ablation lesion gaps were treated with RF energy application. In this section we present our experience performing redo AF ablation procedures in case of AF or atrial flutter recurrence with the simultaneous use of Intellamap Orion catheter with Opal HDx mapping system and pulsed field ablation with Farawave PFA catheter as well as the use of novel lattice tip catheter (Affera mapping and ablation system).

Especially, the following cases describe the successful application of PFA in redo atrial fibrillation procedures post AF or atrial flutter recurrence. In the first four cases an Intellamap Orion multipolar high-resolution mapping catheter was used with Opal HDx mapping system to obtain left atrial voltage and activation mapping. After LA mapping the reconnected veins were recognized, and further ablation was performed with Farawave PFA catheter using basket and flower configurations.

**Figure 2:**
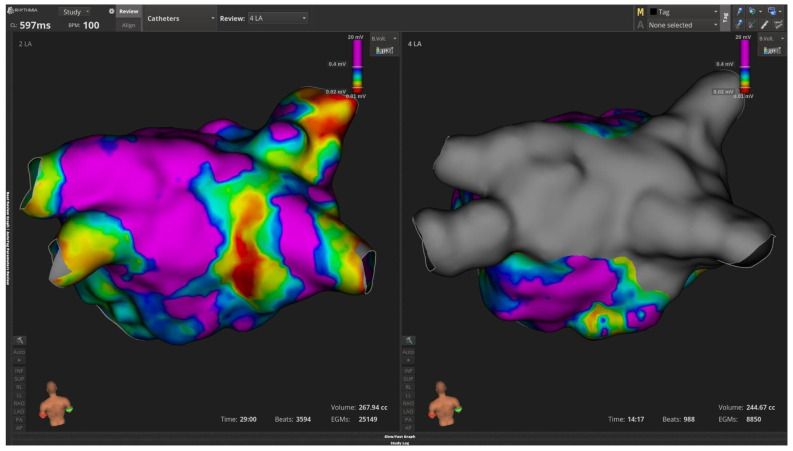
Bipolar (**left**) atrial mapping with Intellamap Orion catheter and Opal HDX system demonstrates reconnection in all 4 pulmonary veins and scar in the posterior wall adjacent to right inferior pulmonary vein. On the (**right**) panel LA voltage map post PVI and posterior wall isolation with Farawave PFA catheter.

**Figure 3:**
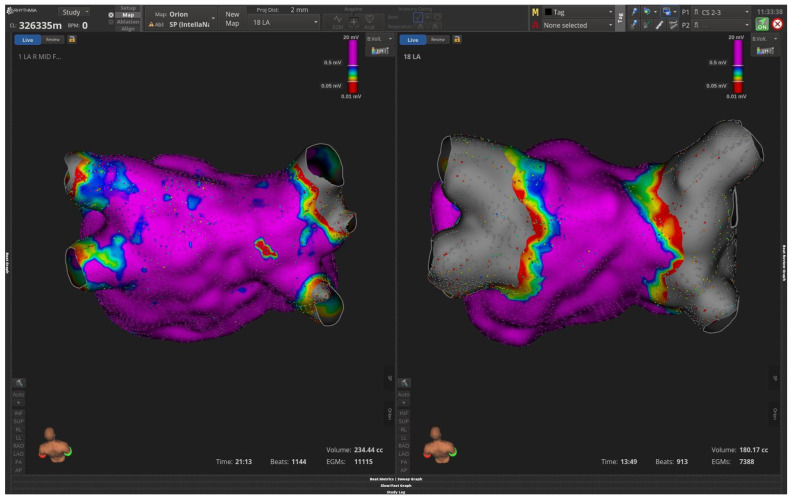
Bipolar LA mapping with Intellamap Orion catheter and Opal HDX system shows ostially isolated (**left**/**right**)-sided pulmonary veins pre-redo PFA. Right panel demonstrates successful wide area circumferential isolation of all PVs (gray area) while the posterior wall is healthy (purple color).

In the first case a 55-year-old male patient with history of paroxysmal AF and previous cryoballoon ablation was admitted electively due to recurrent PAF. Bipolar left atrium mapping with Orion multipolar catheter after successful transeptal puncture revealed ostially isolated left-sided veins along with electrical activity in right PVs. There was also a scar in the posterior wall adjacent to right inferior pulmonary veins. PFA lesions with basket and flower configuration of Farawave catheter successfully re-isolated all 4 PVs along with the posterior wall due to the preexistent scar (Figure 2). The third case refers to a 48-year-old female patient with history of paroxysmal AF and previous successful cryo balloon AF ablation. Mapping with Orion demonstrated ostially isolated LUPV and right-sided veins and reconnected LIPV as depicted in Figure 3. Wide area circumferential ablation PFA lesions were deployed with Farawave PFA catheter, whereas the posterior wall, due to healthy voltages, was decided not to be isolated (Figure 3).

**Figure 4:**
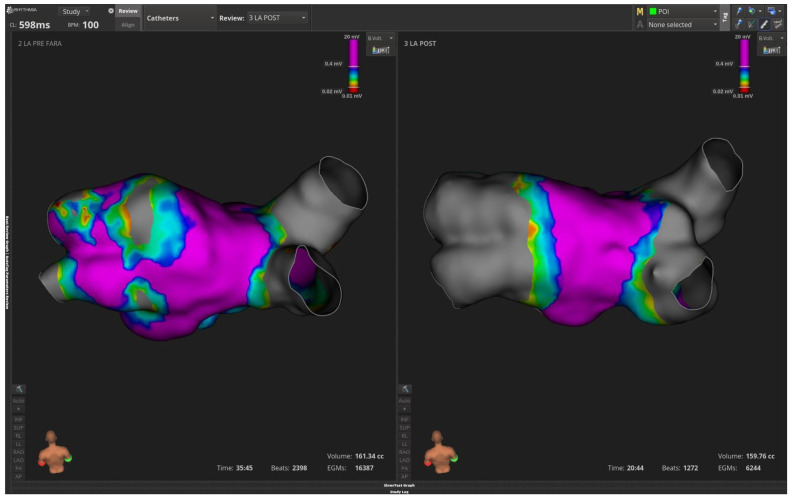
(**Left**) side panel depicts reconnected left-sided veins based on voltage LA mapping with Orion Intellamap catheter. (**Right**) side panel demonstrates low voltage areas in all four pulmonary veins following successful pulmonary vein isolation with the Farawave pulsed field ablation catheter.

**Figure 5:**
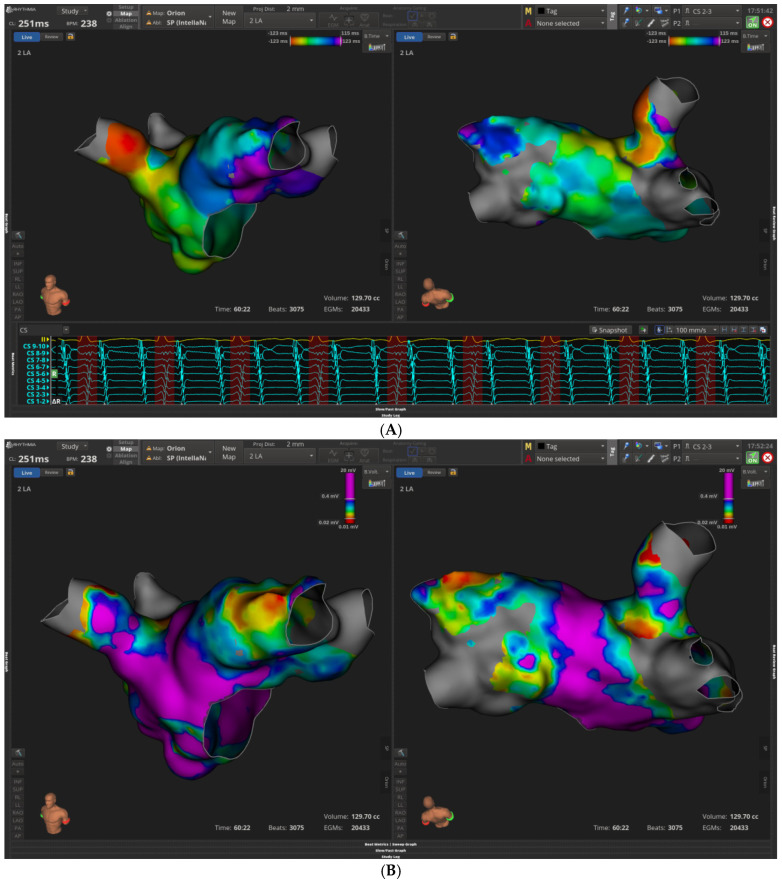

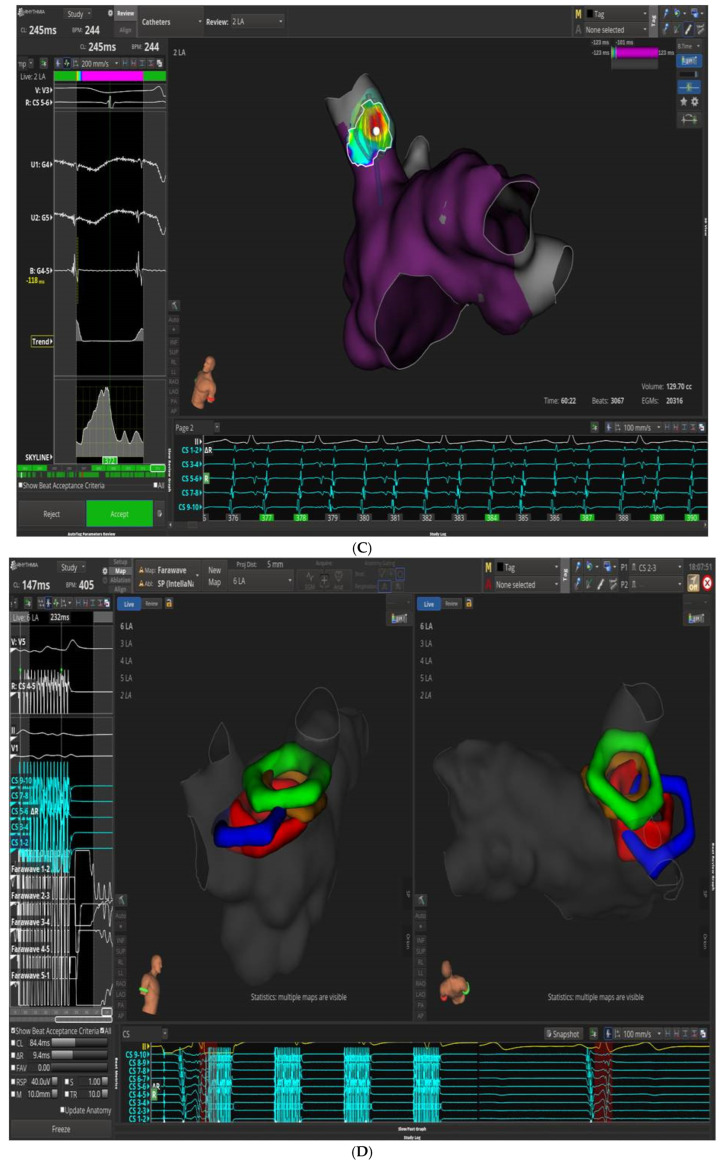
(**A**). Atypical atrial flutter with proximal to distal Cs activation. Earliest activation in RUPV. (**B**). Voltage mapping with Orion Intellamap catheter demonstrates reconnected RUPV and (**C**,**D**). Earliest activation and signal in RUPV and successful termination of atrial flutter with Farawave PFA applications. PFA lesions were represented by the colored rings.

A 39-year-old male with paroxysmal AF and previous successful laser AF ablation was re-admitted due to AF recurrence.LA voltage mapping showed nice isolated right-sided veins but ostially isolated left inferior PV and reconnected LUPV (Figure 4). Again, PFA applications with Farawave PFA catheter re-isolated successfully left-sided PVs as depicted in right panel of Figure 4. A 46-year-old female with a history of paroxysmal AF and previous RF PVI was admitted due to a recurrence of atypical atrial flutter. Decapolar CS activation demonstrated a proximal to distal CS activation but entrainment from cavotricuspid isthmus revealed a long post-pacing interval. As a result, transeptal puncture and LA mapping of atypical flutter with Intellamap Orion wereperformed. Figure 5A,B show the LAT activation mapping during atrial flutter and bipolar voltage mapping of the LA, respectively. Interestingly, the earliest activation point was demonstrated in RUPV as presented in Figure 5C. Further PFA lesions denoted with colored rings were applied and the atypical atrial flutter was successfully terminated. The remaining PVs were isolated. This case represents a safe and straightforward approach during recurrence of atypical atrial flutter with PFA.

**Figure 6:**
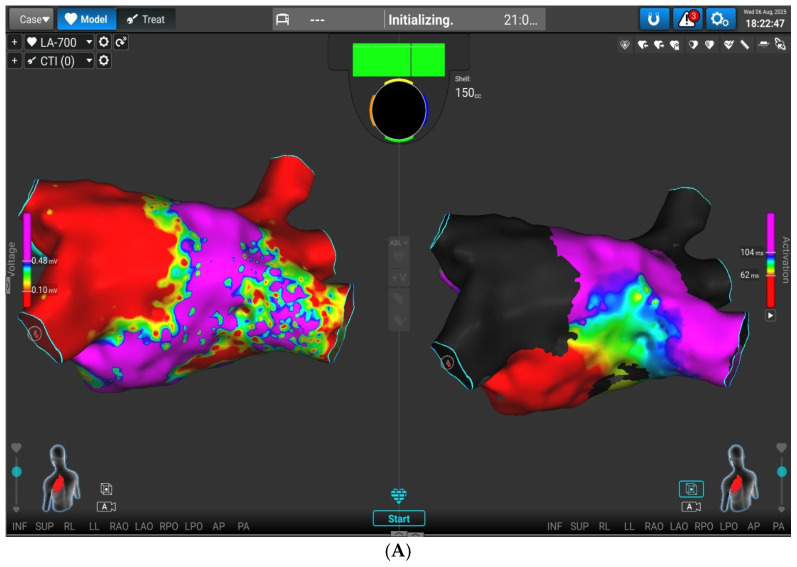

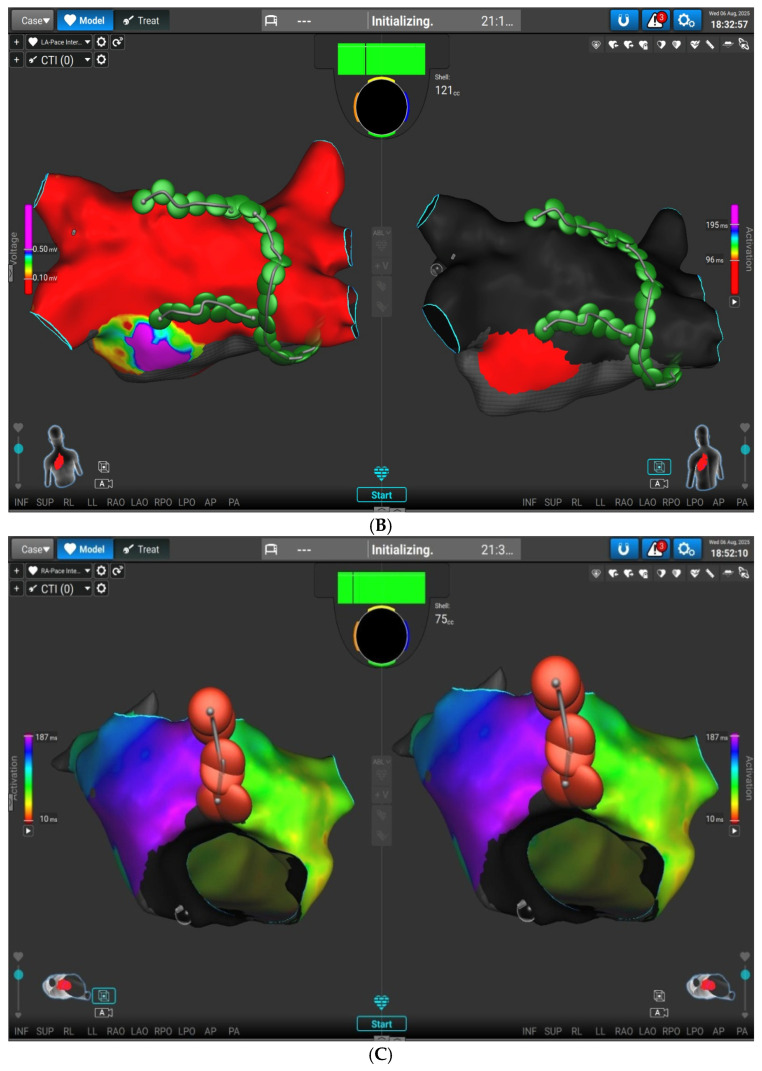
(**A**).Voltage LA mapping with Affera mapping system demonstrates reconnected RIPV with purple voltage. (**B**). Application of PFA lesions (green dots) and isolation of RIPV and posterior wall. (**C**). Previous cavotricuspid isthmus line was connected. Further RF lesions (red dots) were applied, and LAT mapping represents line of block across the isthmus.

Finally, in the last case a 68-year-old lady with frequent recurrences of AF and history of previous cryoballoon ablation combined with RF ablation of typical flutter is presented. In this case LA mapping was performed with the novel lattice catheter and Affera mapping system. Voltage and LAT maps revealed isolated left-sided veins and right upper and middle PV (Figure 6A,B). There was electrical activity in RIPV. Due to frequent paroxysms along with older patient age, RIPV and posterior wall isolation wereperformed with Sphere applying PFA lesions as depicted with green dots. Mapping of the right atrium showed reconnection of the cavotricuspid isthmus line and further RF applications with Affera Sphere established bidirectional block (Figure 6C). The use of the novel Sphere Affera catheter allows rapid LA voltage and activation mapping and dual energy PFA/RF application delivery. One mapping catheter with the possibility of dual ablation lesion capability, thermal and non-thermal, is of paramount importance, leading to reduced procedural laboratory times.

## 4. Discussion

Pulsed field ablation is a promising new modality for the treatment of atrial fibrillation. It achieves efficacy on par with conventional thermal ablation while offering notable safety benefits, particularly by minimizing damage to adjacent structures. Although no clear superiority in long-term outcomes has been established, current data are encouraging with respect to arrhythmia control and patient safety. Further research and extended follow-up are warranted to fully establish the long-term durability of PFA and to define its optimal role in the management of paroxysmal and persistent AF. Notably, PFA catheters integrated with mapping systems can be used for redo atrial fibrillation or de novo atrial flutter cases, establishing a safe, effective and rapid alternative.

PFA is an emerging technology in the field of interventional electrophysiology, and the first reported clinical data indicate efficacy and safety profiles, showing that PFA is growing to become a possible alternative to the so far established treatment modalities, such as RF- or cryoablation [24]. However, data from randomized studies comparing PFA to standard procedures on the matters of recurrence of AF, the lesion durability, and particularly the safety profile are awaited to determine the superiority of PFA to traditional strategies to perform PVI. Further pre-clinical data are required to extend the appliance of the procedure on atrial tachycardias or ventricular arrhythmias. Furthermore, it is yet to be seen whether the current PFA systems are suitable for ablations adjacent to coronary arteries, whereas a recent study analyzed the acute and long-term success of a pentaspline PFA catheter in extra pulmonary vein targets such as coronary sinus isolation, mitral line and left atrial appendage where despite a high acute success isolation rate the 3-month long-term lesion durability remained limited (25). However, this study failed to demonstrate long-term results as it was conducted fluoroscopically with a non-mapping pentaspline catheter. This problem is overwhelmed now with the availability of novel PFA catheters such as Sphere Affera that can perform simultaneously LAT, voltage mapping and point by point ablation either with PFA or RF or both in thick areas such as mitral isthmus.

In conclusion, the latest technology advancements with the incorporation in one catheter of mapping ability as well as dual energy thermal (RF) and non-thermal (PFA) technology can constitute a future alternative in redo AF procedures, reducing the laboratory time as well as the overall cost.

## Figures and Tables

**Table 1:** Summary of major clinical studies evaluating pulsed field ablation for atrial fibrillation.

Study	Design	Endpoints and Follow-Up	Result
**Reddy et al. [7,8]: IMPULSE, PEFCAT, PEFCAT II**	In total the trials included 121 patients where a 12-F over-the-wire PFA catheter was used in order to achieve PVI [7,8].	Evaluation of recurrence of AF, Primary safety events (cardiac tamponade, esophageal injury, phrenic nerve lesions, or brain lesions) Follow-up was accomplished through remapping at 75–90 days after the procedure as well as after 1 year by clinical visit, trans-telephonic monitor, and 24 h Holter to evaluate the recurrence of atrial fibrillation.	Primary safety events: 2.5% (two tamponades); no esophageal, phrenic, or cerebral injury reported. Durable PVI on 75–90-day remapping: 84.8% overall (45.2% monophasic; 83.6% biphasic; 96% optimized biphasic). 12-month Kaplan–Meier freedom from AF recurrence: 81.1 ± 3.8% (84.6 ± 6% with optimized biphasic protocol).
**PULSED AF [9]**	300patients with AF, refractory to the administration of class I or III antiarrhythmic drugs, were enrolled [9].	The primary effectiveness endpoint was freedom from acute procedural failure, arrhythmia recurrence, or antiarrhythmic escalation. The primary safety endpoint was freedom from serious procedure- and device-related adverse events. The follow-up included 1 year via weekly trans telephonic monitoring, EKG at 3-, 6-, and 12- months as well as 6- and 12-month 24 h Holter monitoring, excluding the first three-month recovery period post the procedure.	Acute PVI: 100% of targeted PVs. Major complications: ~0.7%; no reported clinical esophageal, phrenic, or PV injury. Effectiveness broadly consistent with contemporary thermal ablation series; quality of life improved from baseline.
**Cochet H et al. [15]** **Nakatani Y et al. [16]**	41 patients with paroxysmal AF undergoing PVI using either PFA or thermal ablation.	Extra-atrial damages after either PFA or PVI ablation Follow-up within 3 h and 3 months after the procedure.	No phrenic nerve injury documented. Acute esophageal lesions: 43% after thermal ablation; none after PFA. Descending aorta changes: 43% thermal vs. 33% PFA; all resolved by 3 months. PFA produced more homogeneous atrial changes with fewer persistent lesions at 3 months.
**PersAFOne [17]**	25 patients were enrolled in the study, evaluating the application of biphasic, bipolar PFA using a multispline catheter for PVI and left atrial posterior wall (LAPW) ablation under intracardiac echocardiographic guidance.	A composite of major safety events including events occurring within 30 days of the index or reassessment procedures. A remapping was used to assess the lesion durability within 2 and 3 months after the procedure.	Acute PVI: 100% of targeted PVs. Major complications: ~0.7%; no reported clinical esophageal, phrenic, or PV injury. Effectiveness broadly consistent with contemporary thermal ablation series; quality of life improved from baseline.
**InspIRE study [18]**	PFA was applied on 226 participants in order to assess safety and effectiveness of a fully integrated biphasic PFA system with a variable-loop circular catheter for the treatment of drug-refractory paroxysmal atrial fibrillation.	Primary safety, incidence of early-onset primary adverse events, primary effectiveness and confirmed PV isolation with freedom from documented atrial arrhythmia at a follow-up of 12 months.	No phrenic nerve injury documented. Acute esophageal lesions: 43% after thermal ablation; none after PFA. Descending aorta changes: 43% thermal vs. 33% PFA; all resolved by 3 months. PFA produced more homogeneous atrial changes with fewer persistent lesions at 3 months.
**MANIFEST-PF [19]**	1568 patients with paroxysmal or persistent AF undergoing PFA.	The primary effectiveness outcome was freedom from documented AF, atrial flutter or atrial tachycardia. Safety outcomes included the composite of acute (<7 days after the procedure) and latent (>7 days after the procedure) major adverse events. median follow-up of 367 days.	Acute PVI: 100% of targeted PVs. Major complications: ~0.7%; no reported clinical esophageal, phrenic, or PV injury. Effectiveness broadly consistent with contemporary thermal ablation series; quality of life improved from baseline.
**ADVENT [20]**	Study evaluated patients with drug-resistant paroxysmal AF. A total of 305 patients up to 75 years old treated with PFA and 302 with thermal ablation (either radiofrequency or cryoballoon ablation). After discharge was applied oral anticoagulation as well as class I or III antiarrhythmic drugs (amiodarone was excluded).	-Freedom from a composite of initial procedural failure, documented atrial tachyarrhythmia, use of antiarrhythmic drugs, cardioversion, or repetition of ablation after the initial 3- month blanking period. -Freedom from acute and chronic device- and procedure-related serious adverse events [20]. The follow-up was of a total of 12-month period and patients were monitored through 72 h Holter, trans-telephonic electrocardiographic recordings for arrhythmia detection, cardiac computed tomography or MRI scan to assess lesions on pulmonary veins, and in a few participants a brain MRI was performed to assess silent cerebral lesions [20].	No phrenic nerve injury documented. Acute esophageal lesions: 43% after thermal ablation; none after PFA. Descending aorta changes: 43% thermal vs. 33% PFA; all resolved by 3 months. PFA produced more homogeneous atrial changes with fewer persistent lesions at 3 months.
**AdmIRE [21]**	277 patients between 18 and 75 years of age with drug-refractory symptomatic paroxysmal AF who underwent PVI for the first time At least 3 weeks before the procedure, uninterrupted anticoagulation was mandatory.	-Primary safety end points: device- or procedure-related death, major vascular access complications or bleeding, myocardial infarction, pericarditis, heart block, permanent phrenic nerve paralysis, stroke, thromboembolism, transient ischemic attack, pulmonary edema, vagal nerve injury or gastroparesis within 7 days of the procedure, cardiac tamponade or perforation occurring up to30 days after procedure, atrio-esophageal fistula occurring up to 90 days after procedure, and PV stenosis occurring anytime during the 12-month follow-up period. -Primary effectiveness end points: 12-month freedom from documented (symptomatic or asymptomatic) atrial tachyarrhythmia episodes, successful entrance block in allPVs and freedom from ablation	Acute PVI: 100% of targeted PVs. Major complications: ~0.7%; no reported clinical esophageal, phrenic, or PV injury. Effectiveness broadly consistent with contemporary thermal ablation series; quality of life improved from baseline.
**SPHERE Per-AF [22]**	420 patients with persistent AF, who were treated with either ablation using a large-tip catheter with dual pulsed field and radiofrequency energies or ablation using a conventional radiofrequency ablation system	-Primary effectiveness endpoints: freedom from acute procedural failure and repeating ablation at any time within the first year, as well as arrhythmia recurrence, drug initiation or escalation or cardioversion after a 3-month blanking period -Primary safety endpoint: freedom from a composite of serious procedure-related or device-related adverse events	No phrenic nerve injury documented. Acute esophageal lesions: 43% after thermal ablation; none after PFA. Descending aorta changes: 43% thermal vs. 33% PFA; all resolved by 3 months. PFA produced more homogeneous atrial changes with fewer persistent lesions at 3 months.
**DEVI NAIR [23]**	25 patients (paroxysmal or persistent AF), acute PVI was performed under intracardiac echocardiography and 3D mapping guidance, without using fluoroscopy.	Evaluate chronic lesion durability following PVI with a circular PFA catheter (PulseSelect) Invasive remapping immediately after ablation and at 2 months	Acute PVI: 100% of targeted PVs. Major complications: ~0.7%; no reported clinical esophageal, phrenic, or PV injury. Effectiveness broadly consistent with contemporary thermal ablation series; quality of life improved from baseline.

## Data Availability

No new data were created or analyzed in this study. Data sharing is not applicable to this article.
